# Case Report: Successful needle revision of an encapsulated bleb following PAUL glaucoma Implant surgery in a pediatric patient

**DOI:** 10.3389/fopht.2026.1952678

**Published:** 2026-10-09

**Authors:** Abdulrahman Kaki, Jumanah Qedair, Nouf Alzendi, Konrad Schargel

**Affiliations:** 1 Glaucoma Division, King Khaled Eye Specialist Hospital and Research Center, Riyadh, Saudi Arabia; 2 Riyadh Second Health Cluster, Ministry of Health, Riyadh, Saudi Arabia

**Keywords:** bleb needling, encapsulated bleb, mitomycin c, paul glaucoma implant, primary congenital glaucoma

## Abstract

**Claims of priority statement:**

After conducting a literature review in July 2026 through PubMed, Google Scholar, Cochrane, and Web of Science databases using the keywords (Bleb needling; Encapsulated bleb; Mitomycin C; PAUL Glaucoma Implant; Primary congenital glaucoma), we did not find any prior similar reports. Therefore, to the best of our knowledge, this is the first report of successful needling of the PAUL Glaucoma Implant in a child with primary congenital glaucoma.

**Purpose:**

To report the clinical outcomes of a minimally invasive, stepwise transconjunctival bleb needling with mitomycin C (MMC) to rescue a failing, encapsulated PAUL Glaucoma Implant (PGI) in a pediatric patient with refractory primary congenital glaucoma (PCG).

**Methods:**

A case report of a 9-year-old female patient with a complex surgical history, including bilateral deep sclerectomies and PGIs, who presented with uncontrolled left intraocular pressure (IOP) of 27 mmHg despite maximal medical therapy. Baseline B-scan ultrasonography and clinical examination confirmed a +1 encapsulated, loculated fluid collection surrounding the left implant plate. The patient underwent an uneventful, stepwise transconjunctival needle revision using a 27-gauge needle combined with a subconjunctival injection of a 0.3 mL mixture of 0.4 mg/mL MMC and 2% lidocaine to mechanically disrupt the fibrous capsule.

**Results:**

On postoperative day 1, the left IOP was 24 mmHg with a deep anterior chamber. At the 9-day follow-up, the left IOP dropped to 14–17 mmHg with the formation of a quiet, diffuse bleb. By 1.5 months postoperatively, excellent stabilization was achieved with a left IOP of 10 mmHg and best-corrected visual acuity maintained at 20/40 (isolated optotypes reaching 20/30). At the final follow-up (2 months and 11 days), the left IOP remained well-controlled at 13 mmHg. A secondary poor ocular surface with superficial punctate keratitis was successfully managed with aggressive topical lubrication.

**Conclusions:**

Transconjunctival bleb needling augmented with MMC is an effective, conservative alternative to full surgical revision or implant replacement for managing encapsulated, hypertensive drainage blebs following PGI surgery in pediatric patients.

## 1 Introduction

Primary congenital glaucoma (PCG) remains a challenging disease requiring multiple surgical interventions to achieve adequate intraocular pressure (IOP) control and preserve long-term visual function (1, 2). When traditional angle or filtration procedures fail, glaucoma drainage devices (GDDs) serve as an effective therapeutic alternative. Traditional GDDs, such as the valved Ahmed Glaucoma Valve and non-valved Baerveldt Glaucoma Implant, are widely used but carry risks of early hypotony or tube-cornea touch due to larger tube dimensions (3–5). The PAUL Glaucoma Implant (PGI) is a novel non-valved GDD featuring a flexible silicone plate and an ultra-thin tube profile with a smaller internal luminal diameter, designed to promote controlled aqueous filtration while minimizing ocular structural trauma (6–8).

However, a primary mechanism of GDD failure is the formation of a dense, fibrotic encapsulation around the plate, often presenting clinically as a hypertensive phase that limits aqueous filtration (9, 10). While full surgical revision or tube replacement is typically considered, minimally invasive transconjunctival bleb needling combined with an antifibrotic agent, such as mitomycin C (MMC), is a conservative alternative to restore device patency (11, 12).

The novelty of this report lies in demonstrating the successful management and medium-term IOP rescue of a failing, encapsulated PGI using a stepwise transconjunctival needle revision with MMC in a pediatric patient with refractory PCG. We suggest that localized mechanical disruption of the dense fibrous capsule combined with antifibrotic modulation can effectively restore tube patency and aqueous filtration, bypassing the immediate need for a more invasive, full surgical revision. This case highlights the clinical utility and safety of bleb needling as a conservative rescue strategy for failing glaucoma drainage devices in pediatric patients.

## 2 Case description

### 2.1 Patient presentation

A 9-year-old female patient with a known history of bilateral PCG and no known systemic diseases presented with a hypertensive crisis and an encapsulated drainage bleb in the left eye. The patient’s extensive ophthalmic surgical history began in 2017 with bilateral deep sclerectomy (DS).

Her disease progression necessitated several subsequent interventions. For her right eye, she underwent a second DS in 2021, two sessions of micropulse cyclophotocoagulation (MP-CPC) in 2022, two additional MP-CPC sessions in 2023, and the implantation of a PGI in 2023 (with ripcord removal 6 months postoperatively). For her left eye, she underwent a second DS in 2024, followed shortly by a PGI implantation in the same year, with the ripcord successfully removed in 2025.

Before the presentation, the patient’s complex medical regimen consisted of apraclonidine three times daily, brinzolamide/timolol twice daily, and latanoprostene bunod once daily for her left eye. For her right eye, she received bimatoprost once daily. Systemically, she was started on oral acetazolamide 125 mg twice daily.

### 2.2 Diagnostic assessment

Prior biometry from 2023 noted marked axial lengths of 28.25 mm in the right eye (OD) and 26.2 mm in the left eye (OS) (**Figure 1**). A baseline B-scan ultrasonography performed in 2025 confirmed a patent glaucoma drainage tube (**Figure 2**). In the left orbit, an abnormal area of low reflectivity was identified surrounding the tube, consistent with loculated peri-tubular fluid accumulation and bleb encapsulation. Additional structural findings included an elongated, myopic globe secondary to a posterior staphyloma, an almost clear vitreous cavity, and moderate optic disc cupping.

**Figure 1:**
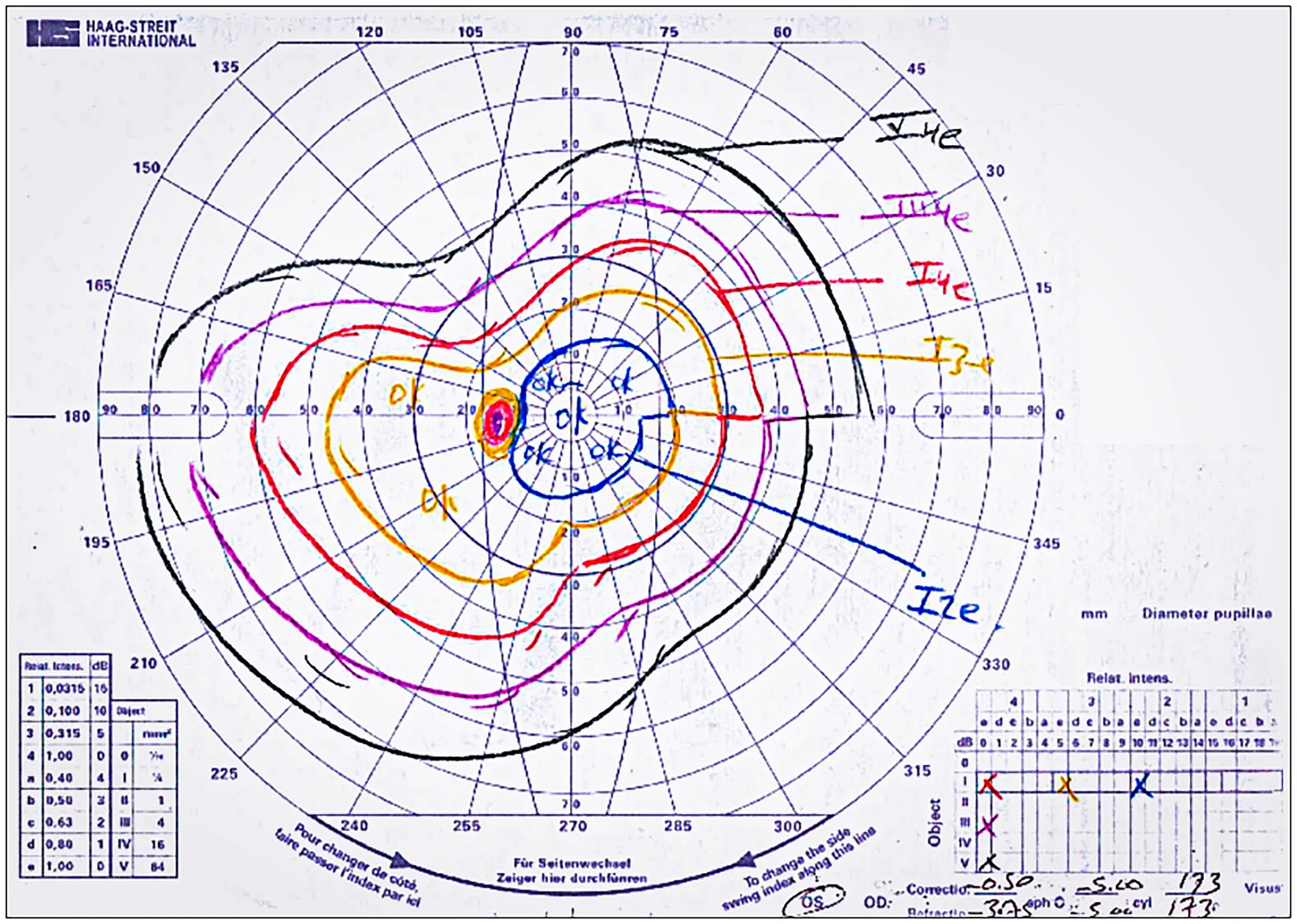
Goldmann visual field of the left eye. It demonstrates a marked concentric contraction of the intermediate and central isopters, indicating a generalized loss of central and paracentral retinal sensitivity typical of advanced glaucoma. A moderately enlarged and vertically elongated temporal blind spot (physiologic scotoma) is present, correlating with the patient’s clinical findings of moderate optic disc cupping (0.65 cup-to-disc ratio), a tilted optic disc, and high axial myopia (axial length: 26.2 mm).

**Figure 2:**
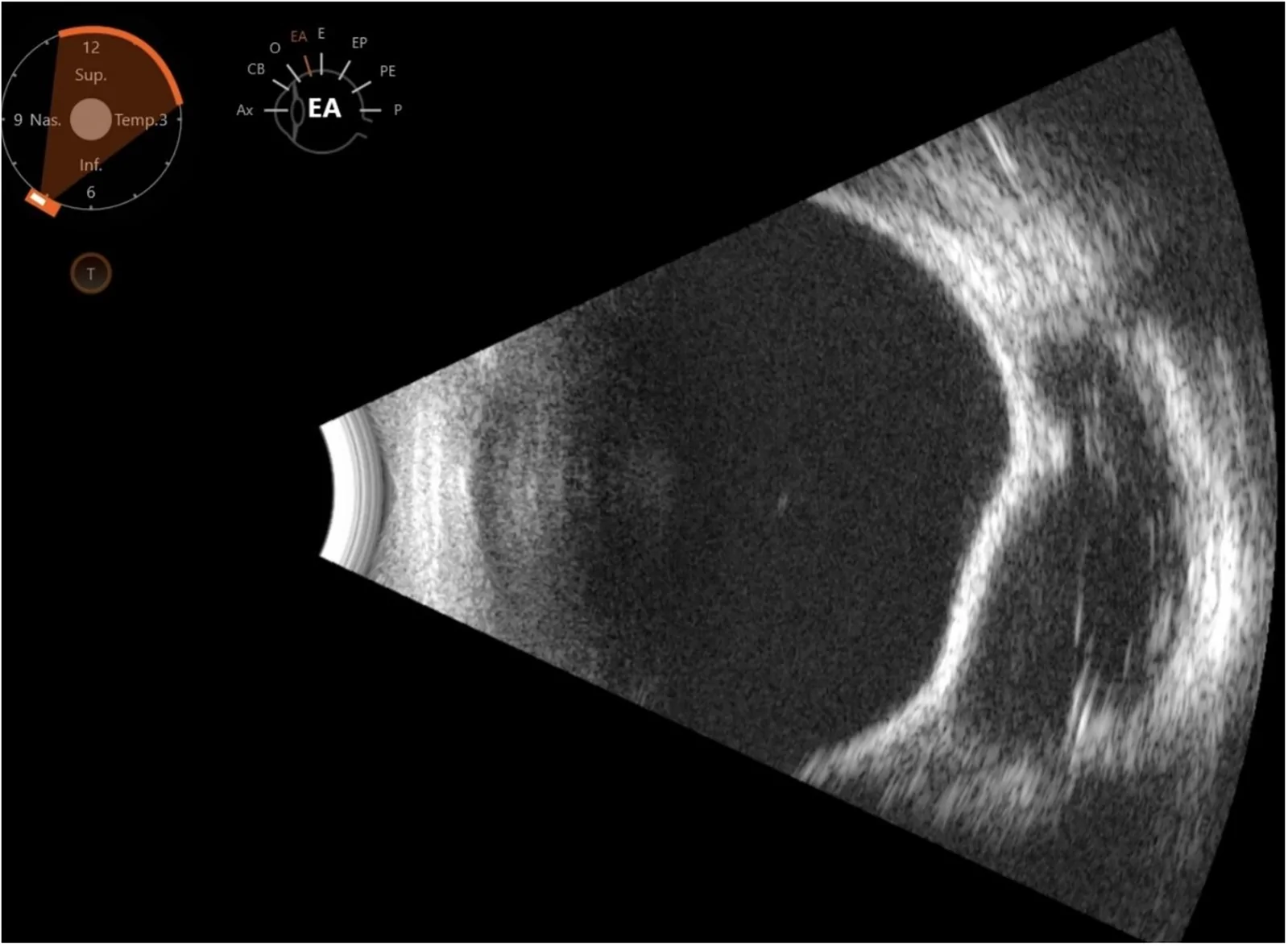
B-scan ultrasonography of the left eye. It demonstrates a patent glaucoma drainage tube with a surrounding abnormal, low-reflectivity area in the orbit, consistent with localized peri-tubular fluid accumulation.

Upon admission for surgical revision of the left eye, the patient’s best-corrected visual acuity (BCVA) was 20/40. IOP measured via iCare tonometry was poorly controlled at 27 mmHg, despite maximum tolerated medical therapy. Slit-lamp examination of the left eye revealed a quiet conjunctiva with a +1 diffuse capsule over a well-covered implant plate. The cornea demonstrated characteristic Haab striae, the lens remained clear, the anterior chamber was deep and quiet, and the optic nerve head demonstrated a cup-to-disc ratio of 0.65 to 0.7.

### 2.3 Therapeutic intervention

In 2026, the patient underwent an uneventful bleb needling with MMC for her left eye under local anesthesia. Following sterile preparation, draping, and speculum placement, the thick, encapsulated capsule was localized. A stepwise needling approach was initiated using a 27-gauge needle. A mixture containing 0.3 mL of 0.4 mg/mL MMC combined with 0.3 mL of 2% lidocaine was injected subconjunctivally into the sub-Tenon’s space. Sweeping mechanical disruption of the fibrous capsule was performed using a Q-tip. At the end of the procedure, subconjunctival dexamethasone was administered, and the eye was dressed with neomycin/polymyxin B/dexamethasone ointment and a protective shield.

### 2.4 Postoperative course and outcomes

On postoperative day 1, the patient’s BCVA remained stable at 20/40 OS. The IOP was 24 mmHg. Slit-lamp examination of the left eye showed mild superior conjunctival injection, a deep anterior chamber, and a well-covered plate with no corneal epithelial defects. The patient was initiated on a fixed-combination dorzolamide/timolol once daily, which was escalated to twice daily following the first postoperative visit (day 9). Her postoperative regimen also included a tapering course of prednisolone acetate drops, ofloxacin drops, and frequent artificial tears.

At the first follow-up (9 days postoperative), the left conjunctiva was quiet with a well-formed, diffuse bleb. The iCare IOP readings had significantly improved, dropping to 14–17 mmHg. Central corneal thickness was recorded at 621 μm. Funduscopic examination revealed a tilted disc with a cup-to-disc ratio of 0.65. By 1.5 months post-needling, the patient demonstrated excellent clinical stabilization. Her BCVA improved to 20/40 (isolated optotypes reaching 20/30). The IOP reached an optimal reading, measuring 10 mmHg. The tube was structurally patent without anterior chamber reaction, and a functioning, diffuse bleb persisted.

At the 2-month follow-up, a transient IOP spike to 22 mmHg was recorded via iCare tonometry, though the bleb morphology remained diffuse and the clinical examination was otherwise unchanged. The IOP returned to a satisfactory baseline just days later (2 months and 11 days postoperatively), with a measured IOP of 13 mmHg. At this final visit, the patient showed signs of a poor ocular surface, characterized by superficial punctate keratitis and multiple pinpoint scars. The treatment plan was adjusted to prioritize aggressive ocular surface lubrication, and the patient was maintained on her current topical hypotensive regimen with strict instructions for regular follow-up.

## 3 Discussion

Managing refractory PCG often requires an escalating surgical strategy, where GDDs like the PGI serve as crucial tertiary interventions (1, 2, 6). Furthermore, PGI has demonstrated particular efficacy in maintaining optimal IOP and reducing the need for antiglaucoma medications in congenital glaucoma, with success rates ranging from 67.2% to 86.6% at the 12-month follow-up (13, 14). However, bleb encapsulation remains a primary mechanism of GDD failure, driving an early or late postoperative hypertensive phase (9, 10). Following implantation, interaction between subconjunctival tissues and aqueous humor enriched with pro-inflammatory cytokines triggers an aggressive wound-healing response (15, 16). Proliferating subconjunctival fibroblasts differentiate into myofibroblasts, depositing excess extracellular matrix and forming a thick, dense, low-permeability collagenous shell around the plate (15, 16). Clinically, this encapsulated wall results in uncompensated IOP elevation.

Managing an encapsulated, hypertensive GDD bleb requires a tailored, stepwise approach. While medical therapy offers a non-invasive initial option, it rarely overcomes the physical resistance of a thick fibrotic shell and carries a risk of ocular surface toxicity (17). Conversely, secondary procedures, such as a second GDD or cyclophotocoagulation, effectively lower IOP but consume limited conjunctival tissue and increase ocular morbidity (18). However, transconjunctival bleb needling with an antimetabolite serves as a minimally invasive strategy, mechanically fenestrating the dense capsule and suppressing fibrosis while preserving tissue for future management (19).

Our case demonstrates that transconjunctival needle revision combined with an antimetabolite can successfully rescue a failing PGI. Similarly, Dibaji et al. (2020) demonstrated the efficacy of transconjunctival needle revision augmented with an antimetabolite for a failed DS in a 14-year-old boy with unilateral congenital glaucoma. In their report, the patient had previously undergone a goniotomy followed by a DS 2 years before, presenting with an IOP of 28 mmHg and a BCVA of 20/70. At the 1-year follow-up following needling, the patient had an IOP of 15 mmHg without antiglaucoma medications. These findings demonstrate that needle revision of failed filtering surgeries is a viable, minimally invasive option in congenital glaucoma (11).

A key strength of this report is the detailed documentation of a stepwise, minimally invasive technique using MMC to successfully rescue an encapsulated, novel PGI in a pediatric patient with PCG, avoiding immediate tube replacement or additional tube placement. However, some limitations must be acknowledged. First, the single-case design limits the generalizability of our findings to broader pediatric populations. Second, the maximum follow-up duration available for this patient was 2 months. While this period captured the critical early post-needling window, including resolution of a transient IOP spike, conclusions regarding long-term efficacy, sustained IOP control, and the late safety profile remain to be established.

In conclusion, while transconjunctival needle revision augmented with MMC appears to be a viable and conservative rescue strategy for failing, encapsulated PGIs in pediatric glaucoma, future prospective studies with larger cohorts and extended follow-up intervals are warranted to establish its long-term durability and safety profile.

## Data Availability

The original contributions presented in the study are included in the article/Supplementary Material. Further inquiries can be directed to the corresponding author.
